# *Subulura brumpti* infection - An outbreak in Japanese quails (*Coturnix coturnix japonica*)

**Published:** 2012

**Authors:** Kumaresan Nagarajan, Desigan Thyagarajan, Muthusamy Raman

**Affiliations:** 1 *Institute of Poultry Production and Management, Tamil Nadu Veterinary and Animal Sciences University, Nandanam, Chennai, India; *; 2 *Department of Veterinary Parasitology, Madras Veterinary College, Tamil Nadu Veterinary and Animal Sciences University, Vepery, Chennai, India.*

**Keywords:** Subulura, Quail Typhilitis, Hemorrhagic Typhilitis, Coturnix

## Abstract

*Subulura brumpti* infection was observed in Japanese quails and caused heavy production loss up to 10%. Gross lesions were confined to caecum of the affected birds. Pathological changes suggestive of acute cecal hemorrhagic enteritis were recorded. Closer observation of the cecal loop revealed wavy movement with thousands of tiny worms. Based on morphometry, the worms were identified as *S. brumpti*. Condition was responded to the albendazole treatment efficiently and all the birds were recovered and production of the flock has been improved.

## Introduction

*Subulura brumpti *(Lopez-Neyra, 1922)^1^ infection occurs in the cecae of fowl, turkey, guinea fowl and wild related birds in Africa, North and South America and Asia.^2^^-^^5^ It come under the family subuluridae; characteristics with mouth of no lips or poorly visible lips. The intermediate hosts are various beetles and cockroaches. *Subulura *infections in fowls are insignificant due to its low pathogenicity. Cuckler and Alicata reported that the caecum showed no evidence of larval penetration or any extensive inflammatory reaction in bob white quail, even though infection could persist as long as eight months.^6^ *Subulura* infection in Japanese quail has been reported by several authors. But its pathologic significance has not been discussed so far. The objective of this study is to present the information regarding hemorrhagic enteritis and production loss in *Coturnix coturnix japonica *affected with *S. brumpti* nematodes.

## Materials and Methods

Four varieties of *C. coturnix japonica *are maintained at Institute of Poultry Production and Management (IPPM) a constituent research unit of Tamil Nadu Veterinary and Animal Sciences University (TANUVAS) since 1982. Population strength of birds were ten thousand numbers of brown variety and one thousand numbers of white, white breasted and golden varieties each are maintained. All of the germplasms have good livability with better egg producing ability and well adapted to local climatic condition of Tamil Nadu. Germplasm seed stocks are sold as parent stock to different hatcheries. Approximately, 200,000 quail chicks per year are sold to commercial quail farming agents in Tamil Nadu. On average, around 2-3% brooder mortality and 0.5% adult mortality are recorded daily. A sudden raise in mortality (11%) over the period of a month was noticed in adult layer birds at the age group of 16 to 18 weeks. Pathological changes suggestive of acute cecal hemorrhagic enteritis were recorded. Cecal loop of the affected birds revealed wavy movement. On opening the affected cecal loop, numerous tiny nematodes with blood mixed contents were noticed. Samples were collected in normal saline for worm identification.

## Results

**Clinical signs. **Affected flock of quails revealed clinical signs such as reduction in feed consumption, lowered egg production around 10%, blood tinged fecal droppings. All affected birds were dull and depressed with ruffled feathers.

**Fig 1:**
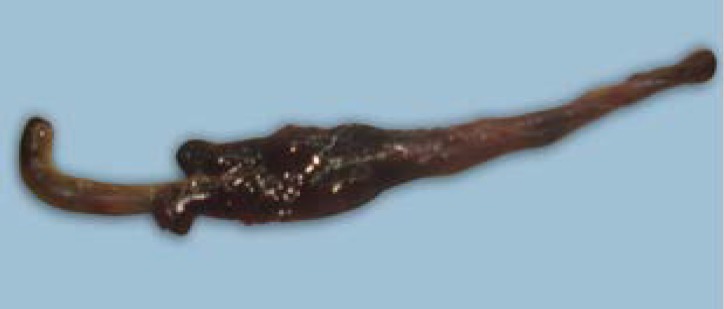
Cecum of the Japanese quail affected by *S. brumpti* infection revealed dark red inflamed area

**Fig 2:**
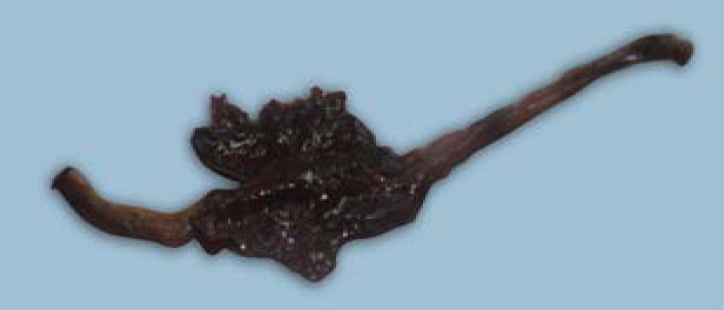
Cecum of the Japanese quail affected by *S. brumpti* infection revealed thousands of tiny worms in the blood mixed content on opening

**Post-mortem findings. **Digestive system except caecum was apparently normal (Fig. 1). Grossly, wave like movement was seen in the caecum. On opening, cecal mucosa was highly inflamed and content was hemorrhagic and blood mixed. On close observation on the contents, revealed thousands and thousands of tiny worms (Fig. 2).

**Fig 3:**
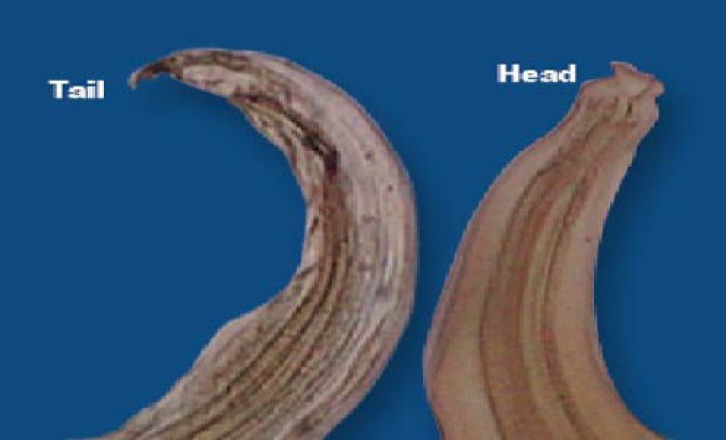
Morphological characterization of head and tail portion of the isolated nematode. 200×

**Worm identification. **Worms were collected and suspended in normal saline, morphological characterization on microscopic examination were recorded. Small nematode with dorsally curved anterior end having hexagonal mouth with six lips bearing median papillae on each was noticed. Males were 6.9-10.0 mm long and 340-420 µm wide, and had a 0.98-1.10 mm long esophagus, lateral alae that extended to the middle of the esophagus, and a tail was curved ventrally. Females were 9.0-13.7 mm long and 460-560 µm wide, with a 1.0-1.3 mm long esophagus, and a straight and conical tail (Fig. 3). Feces content showed thin shelled spherical embryonated eggs with approximate size of 82-86 µm by 66-76 µm. Based on these morphometries, the worms were identified as *S. brumby*.^2^

**Treatment. **Even though these worms are less harmful to the infected birds, production drop affected the performance of the quails. The quails were treated with albendazole in the water at 1 mg kg ^-1^ BW. Since, *S. brumpti *is immediately infective to intermediate hosts, control of the life cycle stages are of utmost importance to control the spread of infections. Hence, measures were taken to avoid entry of aquatic birds to the farm premises and the open water source in and around the farm premise was removed and monitored. Intermediate hosts (cockroaches, beetles, etc.) were destroyed to break the life cycle of *S. brumpti. *Eggs were not found in the feces examined after 15 days post-treatment. Further, regular deworming programme was strictly followed as per the schedule.

## Discussion

In the present study, *S. brumpti* nematode infection was diagnosed in a flock of quails raised in IPPM farm where such an infection had not been reported earlier in Japanese quails. *Subulura* infection in other fowls or in bob white quails produced no evidence of larval penetration or any extensive inflammatory reaction, even though infection could persist as long as eight months.^1^^,^^6^ In contrast, the present study revealed hemorrhagic enteritis in caecum and heavy production loss up to 10% in the Japanese quails affected with *S. brumpti*. The morphological characteristics of the isolated worm were similar to the earlier description of the male and female worms.^2^ The condition was responded to the albendazole treatment efficiently and all the birds were recovered and production of the flock has been improved.
